# Detection and partial discrimination of atypical and classical bovine spongiform encephalopathies in cattle and primates using real-time quaking-induced conversion assay

**DOI:** 10.1371/journal.pone.0172428

**Published:** 2017-02-23

**Authors:** Etienne Levavasseur, Anne-Gaëlle Biacabe, Emmanuel Comoy, Audrey Culeux, Katarina Grznarova, Nicolas Privat, Steve Simoneau, Benoit Flan, Véronique Sazdovitch, Danielle Seilhean, Thierry Baron, Stéphane Haïk

**Affiliations:** 1 Inserm U1127, CNRS UMR 7225, Sorbonne Universités, UPMC Univ Paris VI UMR S 1127, Institut du Cerveau et de la Moelle épinière, Paris, France; 2 Agence nationale de sécurité sanitaire de l’alimentation, de l’environnement et du travail (ANSES), Unité maladies neuro-dégénératives, Lyon, France; 3 Commissariat à l’Énergie Atomique (CEA), Institut des maladies émergentes et des thérapies nouvelles (IMETI), Service d’étude des prions et des infections atypiques (SEPIA), Fontenay-aux-Roses, France; 4 LFB Biomédicaments, Les Ulis, France; 5 AP-HP, Hôpital de la Pitié-Salpêtrière, Neuropathologie, Paris, France; 6 AP-HP, Hôpital de la Pitié-Salpêtrière, Cellule nationale de référence des MCJ, Paris, France; National Institute of Allergy and Infectious Diseases, UNITED STATES

## Abstract

The transmission of classical bovine spongiform encephalopathy (C-BSE) through contaminated meat product consumption is responsible for variant Creutzfeldt-Jakob disease (vCJD) in humans. More recent and atypical forms of BSE (L-BSE and H-BSE) have been identified in cattle since the C-BSE epidemic. Their low incidence and advanced age of onset are compatible with a sporadic origin, as are most cases of Creutzfeldt-Jakob disease (CJD) in humans. Transmissions studies in primates and transgenic mice expressing a human prion protein (PrP) indicated that atypical forms of BSE may be associated with a higher zoonotic potential than classical BSE, and require particular attention for public health. Recently, methods designed to amplify misfolded forms of PrP have emerged as promising tools to detect prion strains and to study their diversity. Here, we validated real-time quaking-induced conversion assay for the discrimination of atypical and classical BSE strains using a large series of bovine samples encompassing all the atypical BSE cases detected by the French Centre of Reference during 10 years of exhaustive active surveillance. We obtained a 100% sensitivity and specificity for atypical BSE detection. In addition, the assay was able to discriminate atypical and classical BSE in non-human primates, and also sporadic CJD and vCJD in humans. The RT-QuIC assay appears as a practical means for a reliable detection of atypical BSE strains in a homologous or heterologous PrP context.

## Introduction

Prion diseases are fatal transmissible disorders affecting humans and animals. These neurodegenerative diseases are characterized by brain vacuolization, neuronal loss and accumulation of PrP^Sc^, an abnormal isoform of the host-encoded cellular prion protein (PrP^c^). The infectious agent is mainly, if not solely, composed of abnormal PrP^Sc^ and is capable of converting cellular PrP^c^ into PrP^Sc^ in an autocatalytical manner [1]. After proteinase K digestion of PrP^Sc^, molecular features of the protease resistant fragment (PrP^res^) can be evidenced by Western blot.

Among animals, the classical bovine spongiform encephalopathy (C-BSE) affects cattle. The C-BSE epidemic in the 1980s became a major matter of concern for human health when the variant of Creutzfeldt-Jakob disease (vCJD) appeared as the result of a C-BSE foodborne transmission to humans [2–4]. Since the C-BSE epidemic, atypical forms of BSE have been reported in cattle [5]. They present a biochemical signature distinct from C-BSE, with a higher (H-BSE) or lower (L-BSE) apparent molecular weight of unglycosylated PrP^res^ observed in Western blot [6]. The annual incidence (1 case per million) and the old age of atypical BSE-affected animals are compatible with a sporadic origin [5]. When transmitted to primates [7–9] and transgenic mice expressing a human PrP [10, 11], L-BSE showed an apparent higher pathogenicity than C-BSE. Pathological and biochemical similarities have been observed between L-BSE in cattle or primates and certain sCJD subtype [7, 12]. When L-BSE was transmitted to sheep [13] and transgenic mice expressing ovine PrP [14, 15], specific strain properties were retained, yet important PrP^res^ molecular changes could be observed in some conditions [16]. Taken together, these studies highlight the importance of a rapid and proper identification of atypical BSE strains in cattle and other species.

Among recent methods developed to amplify prions in vitro, the real-time quaking-induced conversion (RT-QuIC) assay allows the sensitive detection of prion seeding activity in numerous tissues of animal and human origins [17–26]. The RT-QuIC assay relies upon the conversion of recombinant prion protein (recPrP) by prion-associated seeds into amyloid fibrils in presence of an amyloid-sensitive dye, thioflavine T (ThT). The incorporation of ThT within elongating fibrils is monitored in a multiwell plate in real time.

In two recent studies, this test was used successfully for the discrimination of a few C-BSE and L-BSE samples of cattle [27], and for the discrimination of C-, L-, and H-BSE samples of experimentally inoculated cattle [28]. Here, we took advantage of the extensive collection of classical and atypical BSE isolates identified over a 10 year-period of active surveillance in France to validate the rapid discrimination of classical and atypical BSE samples of cattle using RT-QuIC. Furthermore, we were also able to discriminate sporadic CJD and vCJD in humans as reported previously [24, 29], and also classical BSE and atypical L-BSE in primates.

## Materials and methods

### Ethic statement

A written informed consent for autopsy and research use was provided by patient’s relatives, according to the French regulation (L.1232-1 to L.1232-3, Code de la Santé Publique). The brain tissues with the corresponding written informed consent are referred for postmortem diagnosis and research to the French National Neuropathological Network for CJD (funded by the French Government) and to the French National Centre of Reference for prions (funded by the French Institute for Public Health Surveillance). No approval by local ethics committee is required during this procedure.

### Sources of tissues

Samples from primates and cattle existed before the study began. BSE brainstem samples were collected during the active surveillance in France and confirmed by discriminatory western blot by the *Agence Nationale de Sécurité Sanitaire de l’Alimentation*, *de l’Environnement et du Travail* (ANSES, Lyon, France). In this study, 13 H-BSE and 14 L- BSE isolates, encompassing all the atypical BSE cases collected during the period 2000–2010, were analyzed by RT-QuIC. Fifteen C-BSE cases collected during this period were also included. Among samples collected from fallen stock or after several freeze-and-thaw cycles, autolysis was often observed, affecting 10 out of 13 H-BSE samples, 11 out of 14 L-BSE samples and 6 out of 15 C-BSE samples (Table 1). Brainstems from 8 negative BSE animals collected in 2010 were analyzed as negative controls. Brain tissue was taken from 3 non-CJD patients, 3 iatrogenic CJD (iCJD-hGH) patients resulting from an infection with contaminated growth hormone of human origin, 2 vCJD cases (MM2b) and 4 sCJD cases (MM1, MV1, MV2, and VV2) as defined by their *PRNP* codon 129 genotype (MM, MV or VV), their PrP^res^ type (type 1 or 2), and according to the migration pattern of PrP^res^ on Western blot. Patients were referred to the French National Reference Center for Unconventional Transmissible Agents for CJD, and the diagnosis was confirmed biochemically and neuropathologically. Brain samples from cynomolgus macaques previously inoculated via intracerebral and oral routes with classical and atypical L-BSE isolates [7, 30] were analyzed blindly by RT-QuIC.

**Table 1:** Collection of the atypical and classical BSE samples analyzed by RT-QuIC.

Cattle ID	Result of molecular typing by Western blot	Sample shown in Fig 3	Age (year)
01–2437	C-BSE	1	6
01–2579	C-BSE		6
02–2263	C-BSE	2	7
02–2715	C-BSE		8
02–2811	C-BSE	3	6
02–2872	C-BSE		9
02–2992	C-BSE	4	8
04–0881	C-BSE		9
05–0294	C-BSE		9
06–1164	C-BSE	5	12
07–0324	C-BSE	6	12
07–0453	C-BSE		12
09–0170	C-BSE	7	15
09–0335	C-BSE		15
10–0015	C-BSE		6
00–2549	H-BSE		13
01–2604	H-BSE		8
02–0558	H-BSE	8	13
02–2695	H-BSE		11
03–0440	H-BSE	9	16
03–1928	H-BSE		8
03–2095	H-BSE		12
07–0644	H-BSE	10	11
08–0257	H-BSE		18
08–0498	H-BSE	11	8
09–0169	H-BSE		16
09–0497	H-BSE	12	13
10–0161	H-BSE		13
02–2528	L-BSE		8
10–0075	L-BSE	13	15
03–2052	L-BSE	14	13
04–0824	L-BSE	15	19
05–0009	L-BSE		12
06–0931	L-BSE	16	13
07–0012	L-BSE		10
07–1136	L-BSE	17	10
08–0074	L-BSE		11
08–0374	L-BSE	18	15
09–0007	L-BSE		12
09–0397	L-BSE	19	14
09–0481	L-BSE		18
10–0409	L-BSE	20	9

### Recombinant prion protein preparation

Human recombinant full length PrP (codon 129M) (recHuPrP; aa 23–231; GenBank accession number no. M13899) and bovine recombinant full length PrP (recBovPrP; aa 25–242; GenBank accession number no. NP_851358) were purified according to the protocol published previously [31]. Protein concentrations were determined by measuring the absorbance at 280 nm, and aliquots were stored at -80°C until use.

### Brain homogenate preparation

The tissues were prepared in PBS (Sigma-Aldrich) containing 150 mM NaCl, 1 mM EDTA, 0.5% Triton X-100, and Complete Protease Inhibitor Cocktail (Roche) to give a final tissue concentration of 10% (w/v). The homogenization was performed in 2ml centrifuge tubes containing ceramic beads using a FastPrep^®^ 24 instrument (MP Biomedical) for 45 s at speed 6.5. Gross cellular debris were removed from brain homogenates (BH) after a centrifugation at 2000 g for 2 min. Supernatants were collected and stored at -80°C until use.

### Normalization of brain homogenates from affected individuals

To assess the analytical sensitivity of our RT-QuIC assay for the detection of sCJD and determine the minimal amount of detectable PrP^Sc^, brain homogenates (BH) from CJD patients were normalized using known concentrations of recHuPrP. The samples containing PrP^res^ were digested with PK 100μg/ml for 1h at 37°C and loaded onto Novex 4–12% Bis-Tris acrylamide gels (Life Technologies), alongside dilutions of recHuPrP (ranging from 10 to 1.25 ng). To compare the detection of the studied prion strains in the different groups of affected individuals (iCJD patients, non-human primates), brain homogenates were analyzed by Western blot (WB) as previously described [32]. The amount of PrP^res^ in samples was adjusted with a further dilution to match the amount detected in the sample with the lowest PrP^res^ signal at the determined dilution.

After electrophoresis, the separated proteins were transferred to nitrocellulose membrane and immunoblotted with anti-PrP mAb 3F4 (Eurogentec) for human samples. It was followed by an incubation with a secondary antibody coupled with horseradish peroxidase (HRP). The HRP activity was revealed using ECL (GE Biosciences), and the blots were exposed to ECL Hyperfilms (GE Biosciences). The films were scanned using a Bio-Rad GS800 densitometer and analyzed using Bio-Rad Quantity One software. The densities of the single Western blot band corresponding to recHuPrP were compared with the combined densities of the 3 bands corresponding to PrP^res^. To obtain an estimated amount of PrP^res^ in 2 μl equivalent to 100 fg of recHuPrP, the corresponding 100% brain tissue dilutions varied from 10^−6^ (MM1, MV1, MV2) to 5x10^-7^ (MM2b, VV2). For BSE isolates, all the samples were tested by RT-QuIC at the same dilution (10^−4^), regardless of the PrP^res^ level in the sample. A Western blot analysis with TeSeE confirmatory Western blot kit (Bio-Rad) was done to compare relative quantity of PrP^res^ between groups (H-, C- and L-BSE) and to confirm the presence of PrP^res^ when no seeding activity was detected by RT-QuIC.

### RT-QuIC method

The RT-QuIC assay was prepared as described previously [26, 33]. Samples were serially diluted 10-fold in PBS containing 1x N2 media supplement (Life Technologies) and 0.1% SDS. The RT-QuIC reaction mixture was prepared in 1X PBS with final concentration of 300 mM NaCl, 1 mM EDTA, 10 μM thioflavin T, 0.1 mg/ml full length recombinant human (23–231) or bovine (25–241) recPrP, and 98 μl of this mixture were distributed in 96-well black bottom optic plates (Nalgene Nunc). Each reaction was seeded in triplicates with 2 μl of tissue dilution and plates were placed in a BMG Fluostar Omega plate reader (BMG Labtech) at 42°C for 70h (280 cycles, each consisting of 1 min shaking at 600 rpm and 1 min at rest, with ThT fluorescence measurement taken every 15-min with a gain setting of 1000).

### Data analysis

All RT-QuIC experiments were performed at least three times and produced comparable results. At pertinent time points, the statistical significance of the difference between mean fluorescence or lag phases of analyzed groups was assessed using the non-parametric, unpaired t- test with unequal variance (Welch correction), using GraphPad Prism software v6.0 (San Diego, USA).

## Results

### Atypical BSE strains seeded more efficiently the conversion of recombinant PrP than classical BSE strain

**Fig 1:**
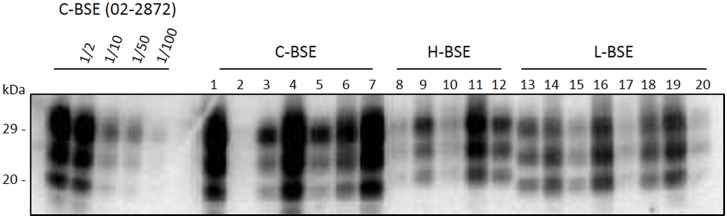
Western blot analysis of PrP^res^ levels in brainstem homogenates from cattle affected by classical or atypical BSE. Homogenates were subjected to proteinase K digestion followed by immunoblotting using Sha31 monoclonal antibody. Dilutions of a C-BSE sample (02–2872) are indicated, along with samples from each C-, H- and L-BSE group. The numbers 1 to 20 refer to cattle identity indicated in Table 1.

**Fig 2:**
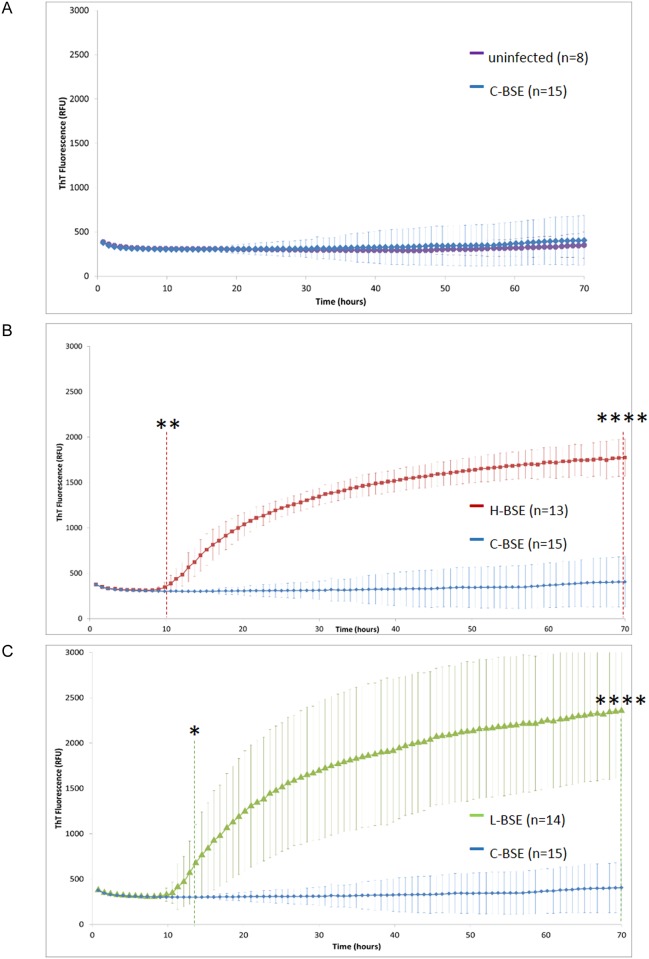
Conversion of human recombinant PrP by atypical BSE strains. Average data and statistical significance of the individual results shown in S1 Fig are represented here. (**A**), classical BSE isolates and uninfected bovine samples. (**B**), classical BSE and atypical H-BSE isolates. (**C**), classical BSE and atypical L-BSE isolates. Each point represents the mean value of 3 replicate relative fluorescence unit readings, which were averaged over the number of animals in each group. Error bars represent the mean standard deviation (SD). Vertical dashed lines indicate a statistically significant difference of signal between the test groups. *, p<0,05; **, p<0,01; ****, p<0,0001.

The seeding efficiency of brain homogenates prepared from cattle affected by atypical and classical BSE was investigated. A total of 15 C-BSE, 14 L-BSE and 13 H-BSE isolates (see details in Table 1), along with 8 negative bovine cases, were analyzed by RT-QuIC. PrP^res^ Western blot results for each BSE group are illustrated in Fig 1. Although a substantial inter-individual variability was observed within each group, a tendency to a higher PrP^res^ level was observed in C-BSE samples. A typical run was performed using 2 plates, in which were included the 8 negative samples and a half of each BSE group. In our conditions, negative and classical BSE samples could not be differentiated using recHuPrP (average data, Fig 2A; individual data, panels A & D in S1 Fig). On the contrary, an efficient amplification of atypical BSE samples was obtained, along with a remarkable homogeneity of the lag phase (~10h) for animals of each group (H-BSE and L-BSE) (Fig 2B and 2C; panels B, C, E, F in S1 Fig). Statistical analyses were performed to determine the time at which the increase in fluorescence becomes significant for each BSE group. No difference was observed between uninfected and C-BSE samples (Fig 2A). The difference of fluorescence signal between C-BSE and H-BSE or L-BSE was statistically significant as early as 10h (p<0.01) (Fig 2B) and 12h (p<0.05) (Fig 2C), respectively, and at terminal 70h time point (p<0.0001).

Experiments were repeated using a different recombinant PrP protein. Although homology of sequence between seed and substrate was described as being not mandatory with RT-QuIC method, we used bovine PrP (recBovPrP) to ascertain that the lack of C-BSE amplification we observed was not due to the use of recHuPrP. Similar results were obtained with recBovPrP. While C-BSE and negative samples remained undistinguishable (panel A in S2 Fig, panels A and D in S3 Fig), H-BSE and L-BSE groups were clearly distinct from C-BSE group (panels B and C in S2 Fig; panels B, C, E and F in S3 Fig). The lag phase was in the same range as that obtained with recHuPrP. The difference of fluorescence signal between C-BSE and H-BSE or L-BSE was statistically significant as early as 11h (p<0.05) (panel B in S2 Fig) and 14h (p<0.05) (panel C in S2 Fig), respectively, and at terminal 70h time point (p<0.0001). We next investigated whether such properties of differential amplification between atypical and classical BSE strains were preserved after passage to non-human primates.

### Efficient amplification of atypical and classical BSE strains after transmission to non-human primates

**Fig 3:**
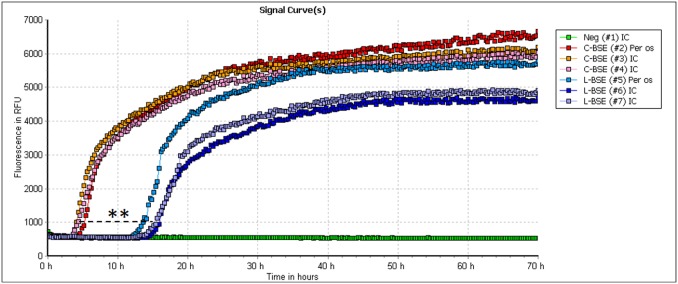
Conversion of human recombinant PrP by C- and L- BSE transmitted to macaques. RT-QuIC reactions were seeded with 10^−4^ and 2.5x10^-5^ dilutions of brain tissue from macaques that had been inoculated by oral (per os) or intracranial (IC) routes with C-BSE and L-BSE, respectively. Each point represents the mean value of 3 replicate relative fluorescence unit readings. Horizontal dashed line indicates a statistically significant difference of lag phase between the L-BSE and C-BSE groups. **, p<0.01.

Western blot showing the PrP^res^ content in brain homogenates from macaques with C-BSE and L-BSE are illustrated in S4 Fig (panel A). Both L-BSE and C-BSE strains, which have been transmitted to non-human primates by intracerebral and oral routes [7,31], seeded efficiently RT-QuIC reactions with human recombinant PrP (Fig 3). An efficient reaction, although with a lower maximum fluorescence level, was also achieved with bovine recombinant PrP (not shown). In both cases, classical BSE samples provided a more efficient seeding material than L-BSE, with a lag phase shorter than 10h. A remarkable homogeneity of the samples within each group was observed, and it was possible to blindly identify 2 groups of samples. No apparent effect of the inoculation route was evidenced. The difference of lag phases (when RFU > 1000) between the 2 groups was statistically significant (p<0.005).

### Brain homogenates from sCJD patients seeded more efficiently the conversion of human recPrP than vCJD brain homogenates

**Fig 4:**
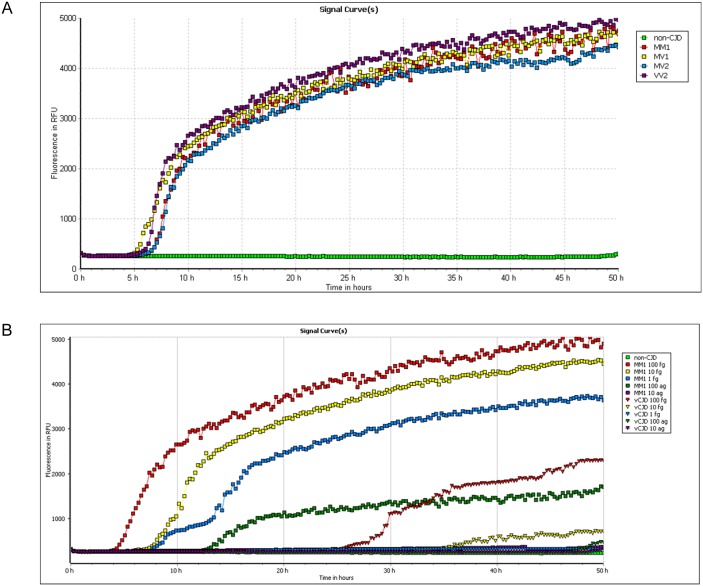
Conversion of human recombinant PrP by sCJD seeds. (**A**) RT-QuIC was seeded with an estimated amount of 100 fg PrP^res^ from MM1, MV1, MV2 and VV2 brain homogenates. Non-CJD brain homogenate was diluted at an equivalent dilution (10^−6^). (**B**) Serial dilutions of sCJD MM1, vCJD MM2b and non-CJD seeds were submitted to RT-QuIC. Amounts of PrP^res^ detected in brain homogenate ranged from the equivalent of 100 fg to 10 ag. Each point represents the mean value of 3 replicate relative fluorescence unit readings.

We next assessed whether differential seeding activity can be observed in humans using seeds from patients infected with the C-BSE agent and from patients with sporadic CJD. Western blot results showing the PrP^res^ content in brain homogenates from sCJD and vCJD patients are illustrated in S4 Fig (panel B). Brain homogenates from sCJD patients with different molecular subtypes (MM1, MV1, MV2 and VV2) seeded with the same efficiency RT-QuIC reactions using full-length recHuPrP (Fig 4A), after the normalization of the PrP^res^ levels. The assay was able to detect the molecular subtype MM1 down to a 10^−9^ dilution of 100% brain tissue, corresponding to 100 ag of PrP^res^ (Fig 4B). In contrast, and despite equivalent amounts of PrP^res^ (100 fg) used to seed the reaction, vCJD brain homogenates proved less efficient to initiate RT-QuIC reactions, and only a sensitivity down to a 10^−7^ brain dilution (equivalent to 10 fg of PrP^res^) could usually be achieved in our conditions (Fig 4B). Replacing human recPrP with bovine recPrP did not improve the detection of vCJD (data not shown). Negative results were obtained with normal brain homogenates (NBH), which were diluted 10^6^ fold starting from 100% brain tissue.

### Brain homogenates from iCJD-hGH patients seeded efficiently the conversion of human recPrP

**Fig 5:**
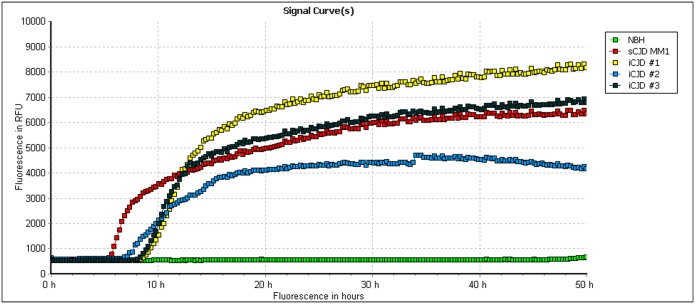
Conversion of human recombinant PrP by iCJD seed. RT-QuIC reactions were seeded with 10^−6^ and 8x10^-7^ dilutions of brain tissues from one sCJD MM1 and three iCJD-hGH patients (iCJD #1, #2 and #3), respectively. Each point represents the mean value of 3 replicate relative fluorescence unit readings.

We also studied the efficiency of our RT-QuIC assay with iatrogenic CJD, an additional infectious form of human prion disease due to a contamination by the peripheral route, after cadaver–derived human growth hormone treatment. Brain homogenates from 3 French iatrogenic cases seeded as efficiently as sCJD MM1 the conversion of human recPrP, as sporadic and iatrogenic seeds were indistinguishable and showed similar lag-phases (<10h) (Fig 5).

## Discussion

In this study, we assessed the power of discrimination of BSE strains from cattle using RT-QuIC. Indeed, in two recent studies, this assay was used successfully for the discrimination of a few C-BSE and L-BSE samples from cattle [27], and for the discrimination of C-, L-, and H-BSE samples from experimentally inoculated cattle [28]. Here, we took advantage of the extensive collection of classical and atypical BSE isolates identified over a 10 year-period of active surveillance in France to validate RT-QuIC performances through the analysis of 15 classical and 27 atypical BSE samples from cattle. Classical BSE, from which human vCJD is derived, was particularly inefficient to seed recombinant PrP and could not be differentiated from negative bovine samples. On the contrary, all the atypical BSE samples were readily detected, with the same lag phase, despite very different PrP^res^ content. Thus, our study confirms previous results obtained with 5 L-BSE and 4 C-BSE natural cases by Orru et al [27] and with 3 experimental cases of each BSE subtype by Masujin et al [28], using large series of natural cases (15 C-BSE, 14 L-BSE and 13 H-BSE cases). In addition, we further tested the detection of L-BSE and C-BSE after transmission to macaques and compared the seeding properties of vCJD samples to those obtained with sCJD and French iCJD after GH treatment.

Atypical cases of BSE putatively represent sporadic forms of prion disease in cattle, with PrP^sc^ glycotypes, neuropathology and PrP^sc^ deposition different from those observed in classical BSE [5, 12]. In humans, we also observed a differential amplification between seeds from sCJD and vCJD, as previously reported [24]. Using sCJD brain homogenates, we achieved a sensitivity in a similar range to that reported by others using hamster [24] or human recPrP substrates [18], without false positive reaction, contrasting with the experience from other laboratories [21]. Likewise, vCJD homogenates were less efficient to seed reactions than sCJD homogenates. Possible explanations have been discounted, such as case-to-case variations, brain region variation, or inhibitors present in vCJD brain [24]. In our study, we tested different regions, from 2 different vCJD patients, without any improvement of the detection.

RT-QuIC discrimination may rely on differences in abnormal PrP assemblies sustaining distinct seeding activities that may vary with several factors (sporadic or infectious origin of the disease, affected species, route of infection). We were able to discriminate brain samples from macaques inoculated with C-BSE and with L-BSE. Surprisingly, in macaques, C-BSE showed the most efficient seeding activity while it was relatively inefficient in humans. Differences in PrP amino acid sequence might contribute to this discrepancy. Mature forms of bovine and human PrP share 91.3% of amino acid sequence, and bovine and cynomolgus macaques PrP only 88.7%. Different regions of PrP have been proposed as key domains for fibrillization, such as the S1H1S2 region [34, 35] or the H2H3 domain [36]. It was suggested that single amino acid variations in the H2 and H3 domains trigger different oligomerization pathways [37]. A sequence alignment of bovine and macaque PrP amino acids shows variations at residues 100 and 108 in macaque (and not in human PrP). This central region of PrP contains an amyloidogenic sequence AGAAAAGA that appears critical for PrP^res^ formation [38] and because corresponding synthetic peptides are able to specifically inhibit *in vitro* formation of PrP^res^ [39]. Other variations exist in macaque PrP at residue 143 (H1 domain), residue 155 (6 amino acids before S2 domain), and residue 220 (H3 domain). Hence, it is possible that such amino acid variations in critical regions of the macaque PrP has an impact on the quaternary structure of PrP^sc^ assemblies and therefore on the related seeding activity. For example, the H187R mutation in the human PrP has a dramatic effect on the protein folding, resulting in a markedly increased propensity to oligomerize [40].

However, the unpredictable manner of the biological changes of prion strain properties during interspecies transmission has been extensively described [41–44]. In more recent studies, when L-BSE isolates were propagated in wild type mice [45] and transgenic mice expressing ovine PrP [15], strain features closely similar to those of C-BSE agent were observed. In addition, a shift in the biochemical signature of the C-BSE agent was observed in the spinal cord of orally-infected macaques at the preclinical stage [46]. Altogether, these data highlight the fact that the properties of BSE strains may evolve during interspecies transmission, notably in macaques. Our results suggest that such a strain variation also has an impact on seeding activity.

A main feature of C-BSE-infected brain tissue from various animal models including macaques is the presence of amyloid plaques [47–52] composed of PrP^Sc^ fibers which could modulate the seeding activity of these tissues. However, plaques are also observed in the brain of vCJD patients, and vCJD brain homogenates show a poor seeding activity. Another characteristic of C-BSE in macaques is the high proportion of diglycosylated PrP^res^, but C-BSE or vCJD PrP^res^ share the same feature, and yet are not associated with an efficient seeding activity. Likewise, the Western blot type of PrP^res^ as defined by the molecular mass of the unglycosylated PrP^res^ after proteinase K digestion cannot account for these different seeding activities, since PrP^res^ type 2 is distributed in both groups with efficient (sCJD MV2, VV2, C-BSE in macaques) and inefficient (vCJD) seeding properties. It was intriguing that PrP^sc^ from all the studied natural diseases with a sporadic or presumably sporadic origin (all sCJD subtypes, all atypical BSE isolates) showed a high level of seeding activity, unlike peripherally acquired diseases due to the C-BSE agent (vCJD, C-BSE). To investigate the role of the peripheral route on the selection of PrP^Sc^ species with seeding activity, we analyzed brain homogenates from iatrogenic CJD cases secondary to growth hormone treatment, which are acquired prion diseases and result from a human-to-human CJD transmission. We found similar and efficient seeding activities for sCJD MM1 and iCJD-hGH cases using RT-QuIC. Moreover, we also found similar and efficient seeding activities in brain homogenates from non-human primates inoculated intracerebrally and via the oral route with C-BSE or L-BSE. Altogether, our data do not support the peripheral route as a main factor influencing the selection of PrP^sc^ species with low seeding activity. In our hands, it appears that this poorly efficient RT-QuIC profile was limited to the C-BSE agent, which propagated in cattle and humans.

To conclude, we showed that RT-QuIC detects PrP^Sc^ from a large series of 27 atypical BSE isolates within hours, and provides a promising tool for the diagnosis of these natural diseases and for their discrimination from C-BSE in a homologous and heterologous PrP context.

## Supporting information

S1 Fig Efficient conversion of human recombinant PrP by atypical BSE strains. RT-QuIC reactions were seeded with 10^−4^ dilutions of bovine tissue (brainstem), using human recombinant protein. (**A**) and (**D**), classical BSE and uninfected bovine tissues. (**B**) and (**E**), classical BSE and atypical H-BSE. (**C**) and (**F**), classical BSE and atypical L-BSE. Each point represents the mean value of 3 replicate relative fluorescence unit readings.
(PDF)

S2 Fig Conversion of bovine recombinant PrP by atypical BSE strains. Average data and statistical significance of the individual results presented in S3 Fig are represented here. (**A**), classical BSE isolates and uninfected bovine samples. (**B**), classical BSE and atypical H-BSE isolates. (**C**), classical BSE and atypical L-BSE isolates. Each point represents the mean value of 3 replicate relative fluorescence unit readings, which were averaged over the number of animals in each group. Error bars represent the mean standard deviation (SD). Vertical dashed lines indicate a statistically significant difference of signal between the test groups. *, p<0,05; ****, p<0,0001.
(PDF)

S3 Fig Conversion of bovine recombinant PrP by atypical BSE strains. RT-QuIC reactions were seeded with 10^−4^ dilutions of bovine tissue (brainstem), using bovine recombinant protein. (**A**) and (**D**), classical BSE isolates and uninfected bovine samples. (**B**) and (**E**), classical BSE and atypical H-BSE isolates. (**C**) and (**F**), classical BSE and atypical L-BSE isolates. Each point represents the mean value of 3 replicate relative fluorescence unit readings.
(PDF)

S4 Fig Western blot analysis of PrP^res^ content in brain homogenates (A) from non-human primates inoculated with classical or atypical L-BSE and (B) from human sCJD and vCJD patients. Homogenates were subjected to proteinase K digestion and serial dilutions were detected by immunoblotting using Sha31 (primates) or 3F4 (CJD patients) monoclonal antibodies.
(PDF)
