# Supplementary figures and images for: Detection and partial discrimination of atypical and classical bovine spongiform encephalopathies in cattle and primates using real-time quaking-induced conversion assay
Source: PLoS One. 2017 Feb 23;12(2):e0172428. doi: 10.1371/journal.pone.0172428 (PMC5322914; doi:10.1371/journal.pone.0172428)

S1 Fig

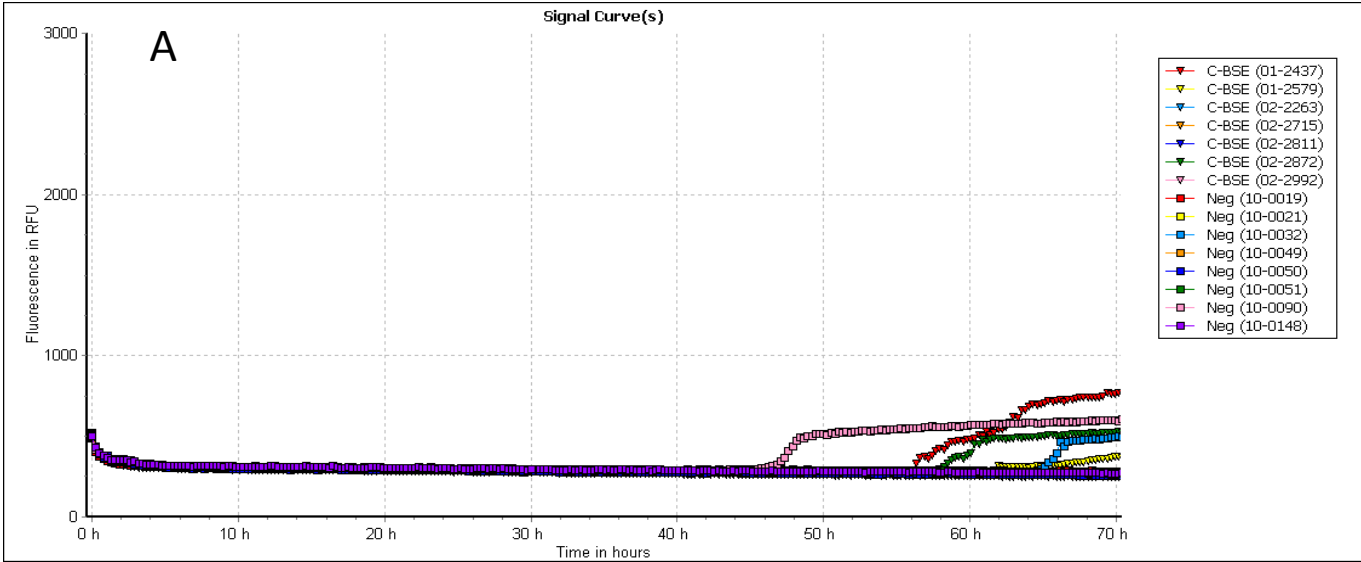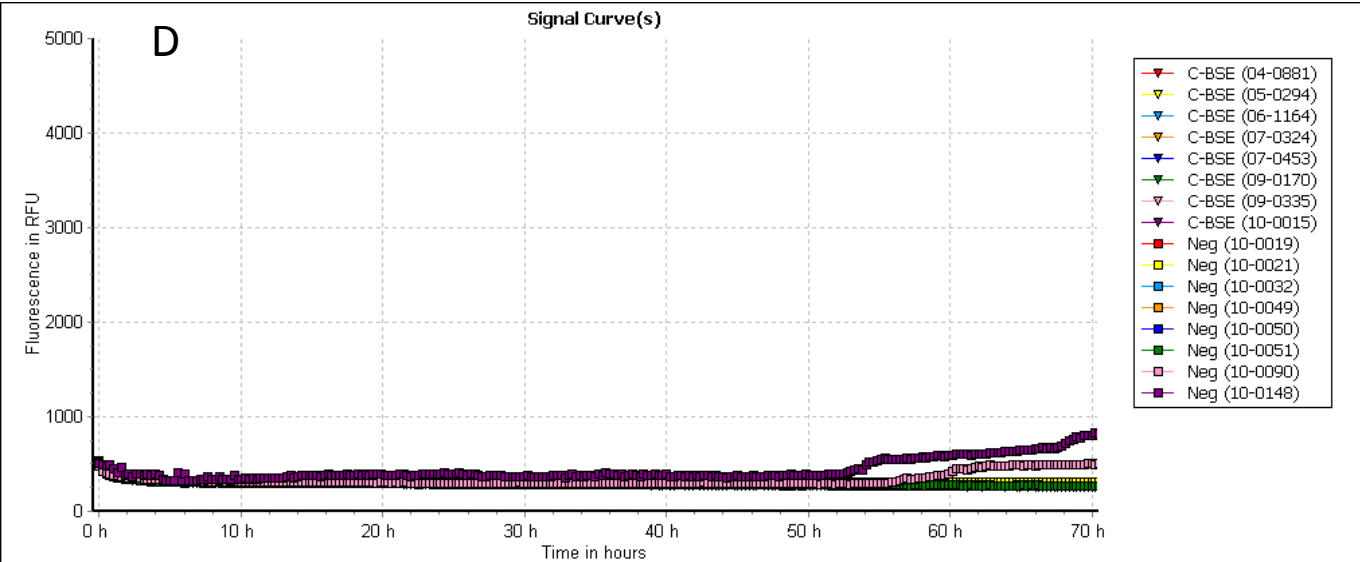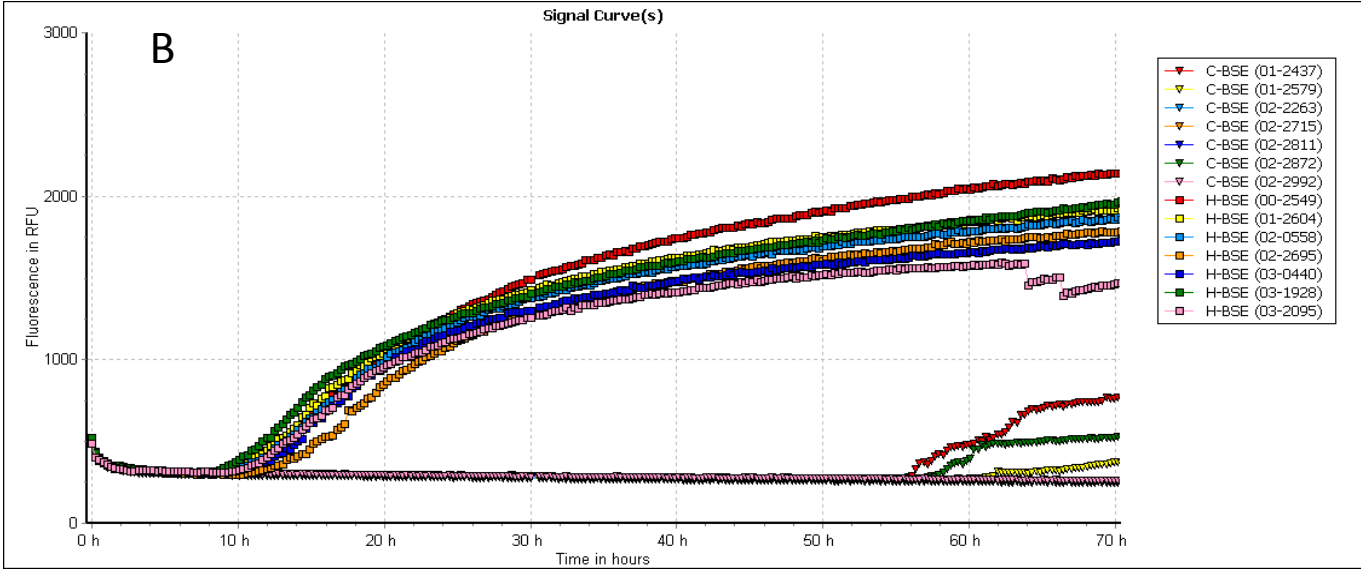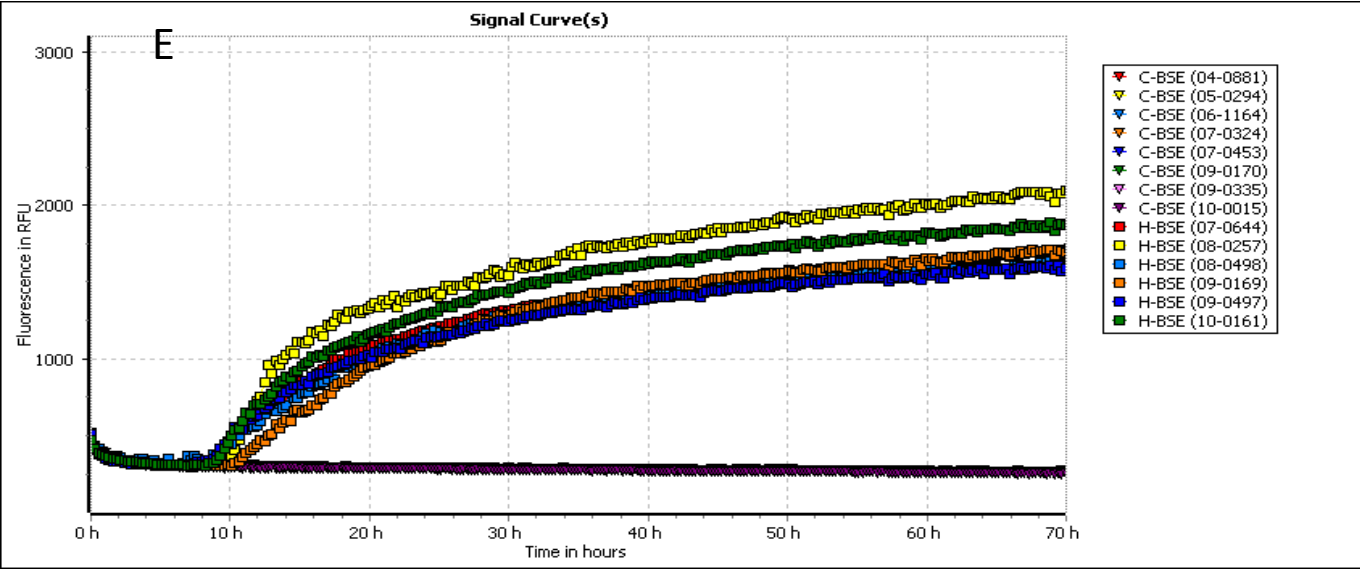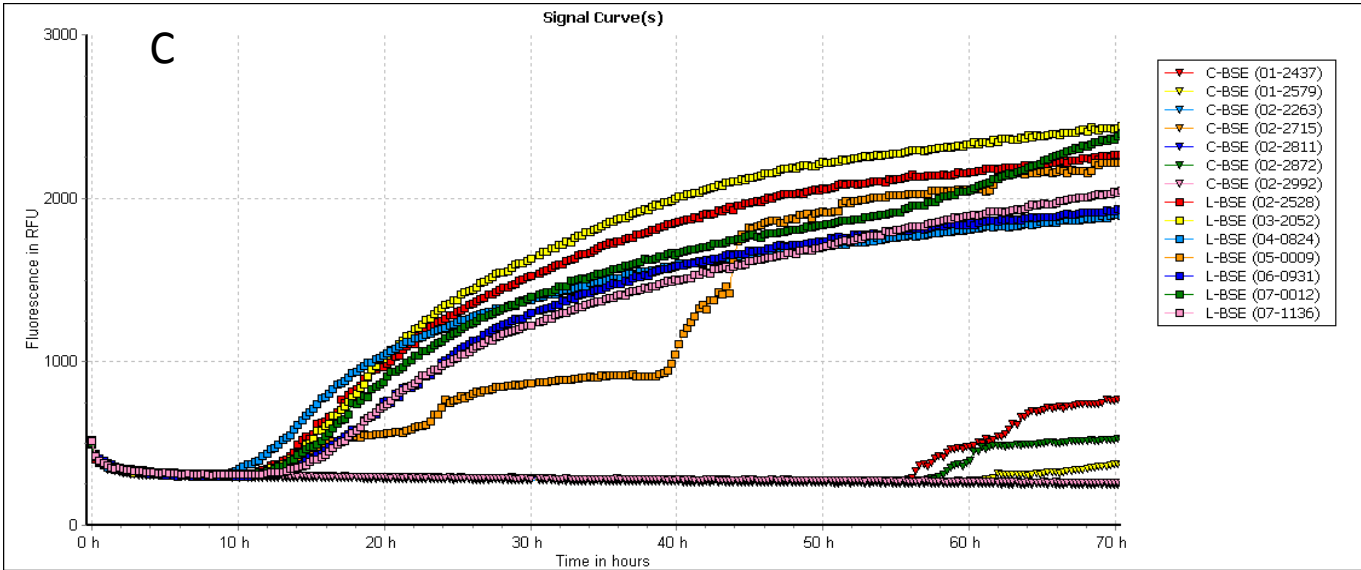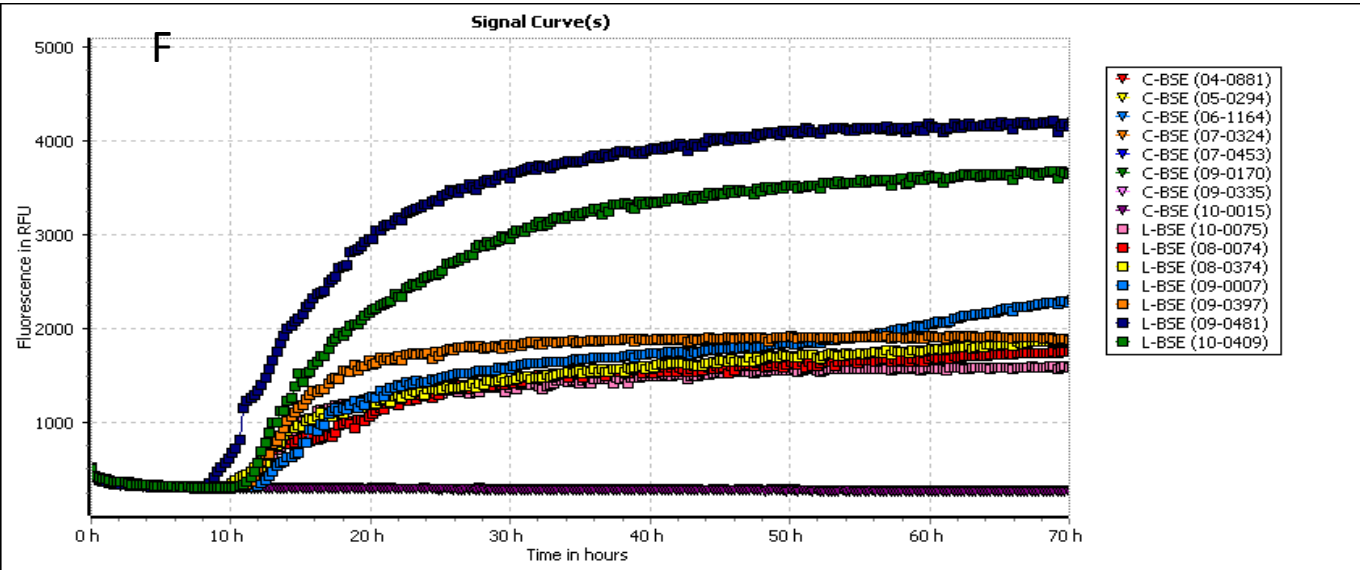

Supplement: S1 Fig — RT-QuIC reactions were seeded with 10−4 dilutions of bovine tissue (brainstem), using human recombinant protein. (A) and (D), classical BSE and uninfected bovine tissues. (B) and (E), classical BSE and atypical H-BSE. (C) and (F), classical BSE and atypical L-BSE. Each point represents the mean value of 3 replicate relative fluorescence unit readings. (PDF) [file pone.0172428.s001.pdf]

S2 Fig

A

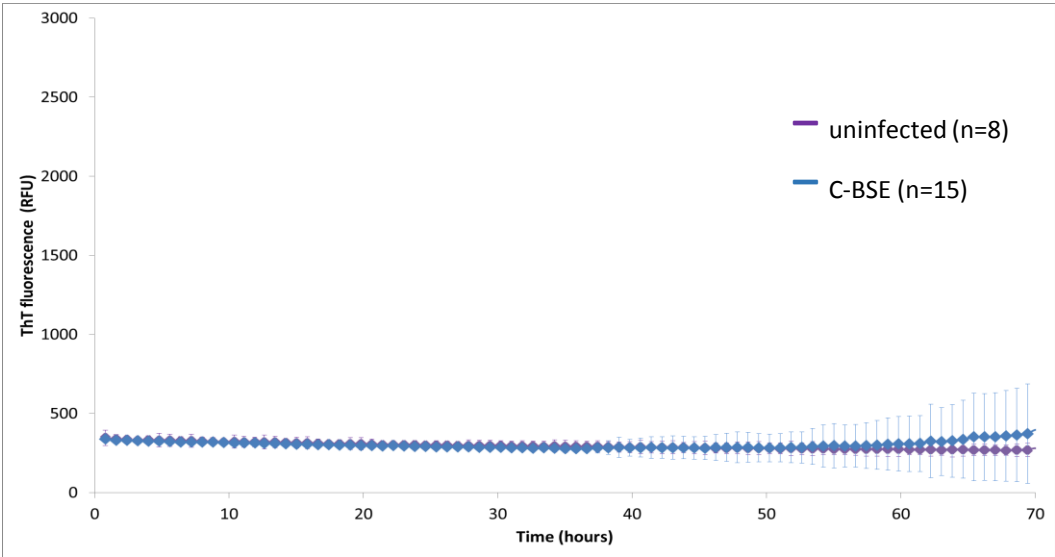

B

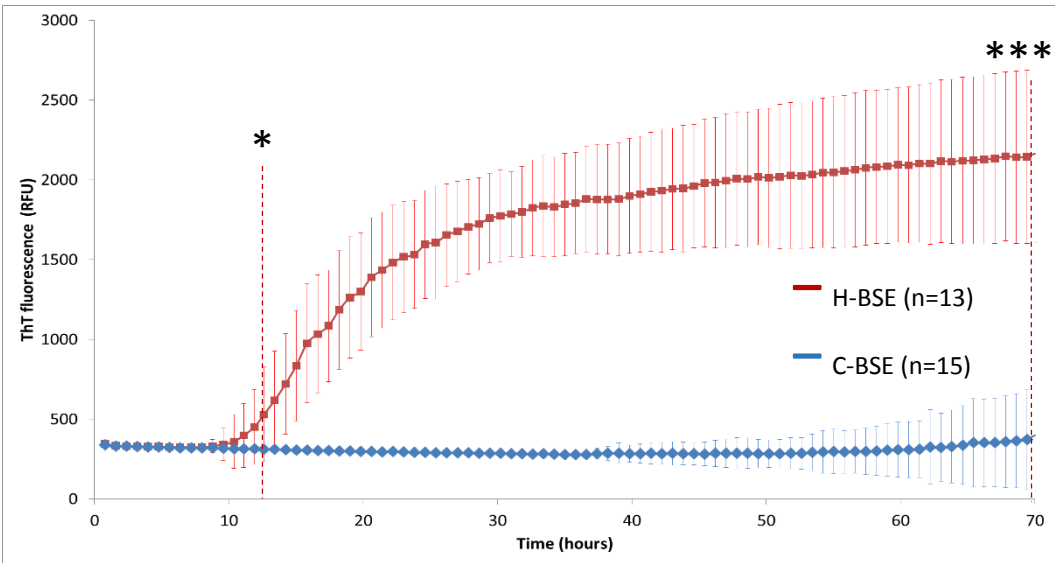

C

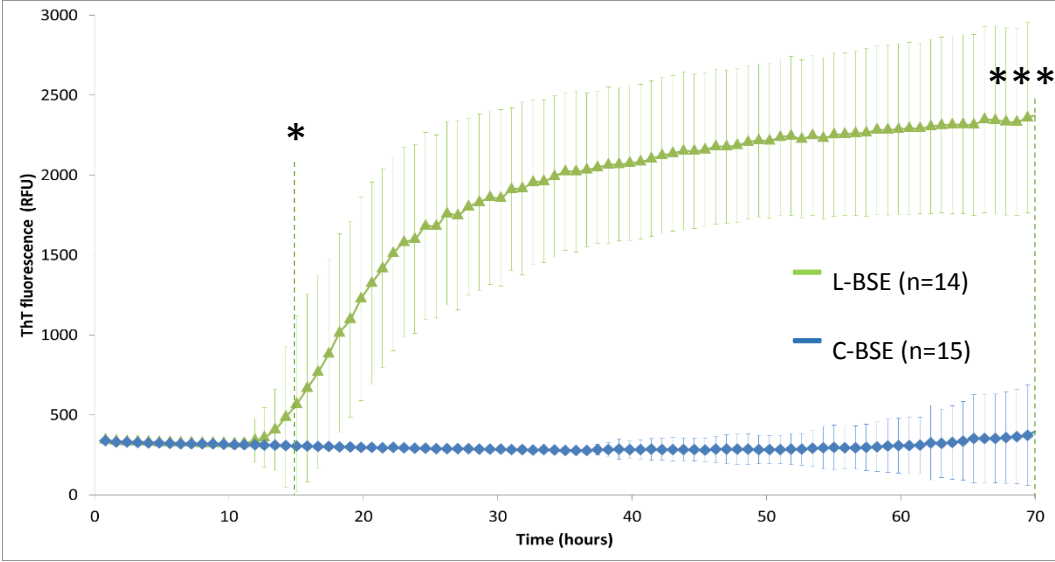

Supplement: S2 Fig — Average data and statistical significance of the individual results presented in S3 Fig are represented here. (A), classical BSE isolates and uninfected bovine samples. (B), classical BSE and atypical H-BSE isolates. (C), classical BSE and atypical L-BSE isolates. Each point represents the mean value of 3 replicate relative fluorescence unit readings, which were averaged over the number of animals in each group. Error bars represent the mean standard deviation (SD). Vertical dashed lines indicate a statistically significant difference of signal between the test groups. *, p<0,05; ****, p<0,0001. (PDF) [file pone.0172428.s002.pdf]

S3 Fig

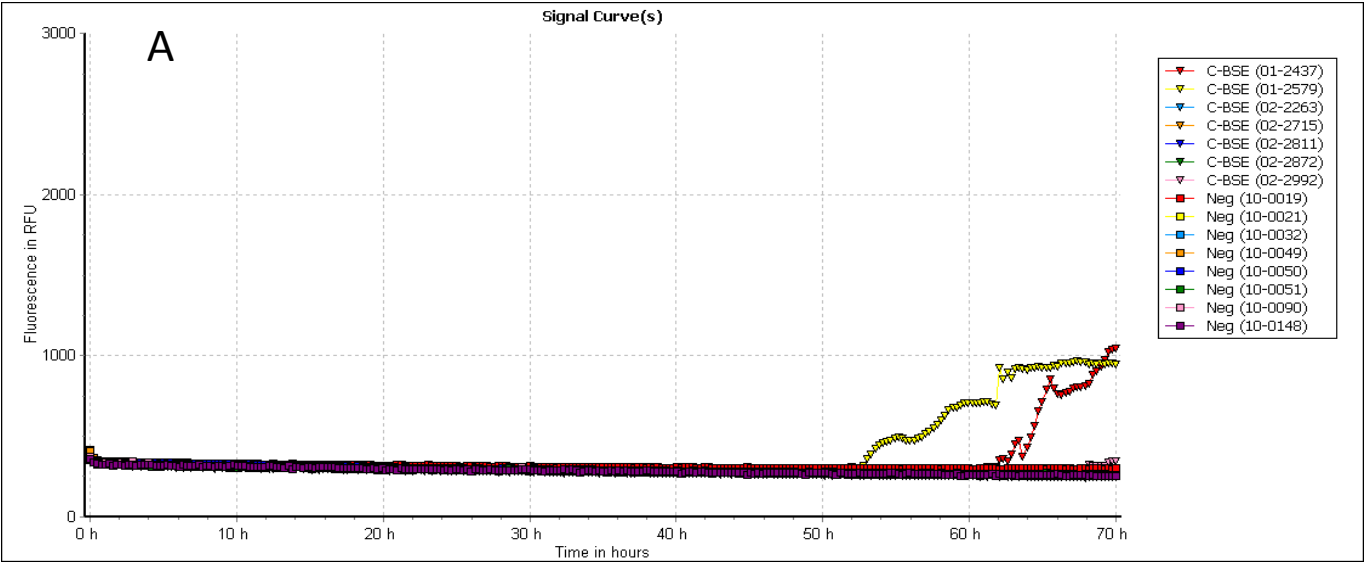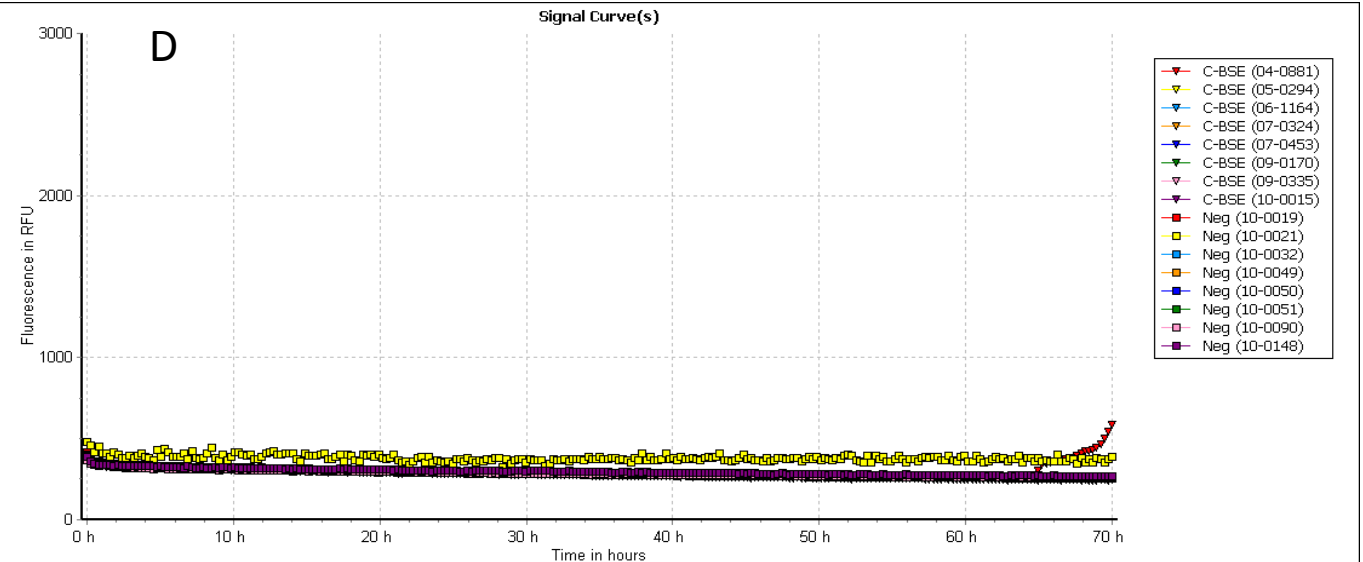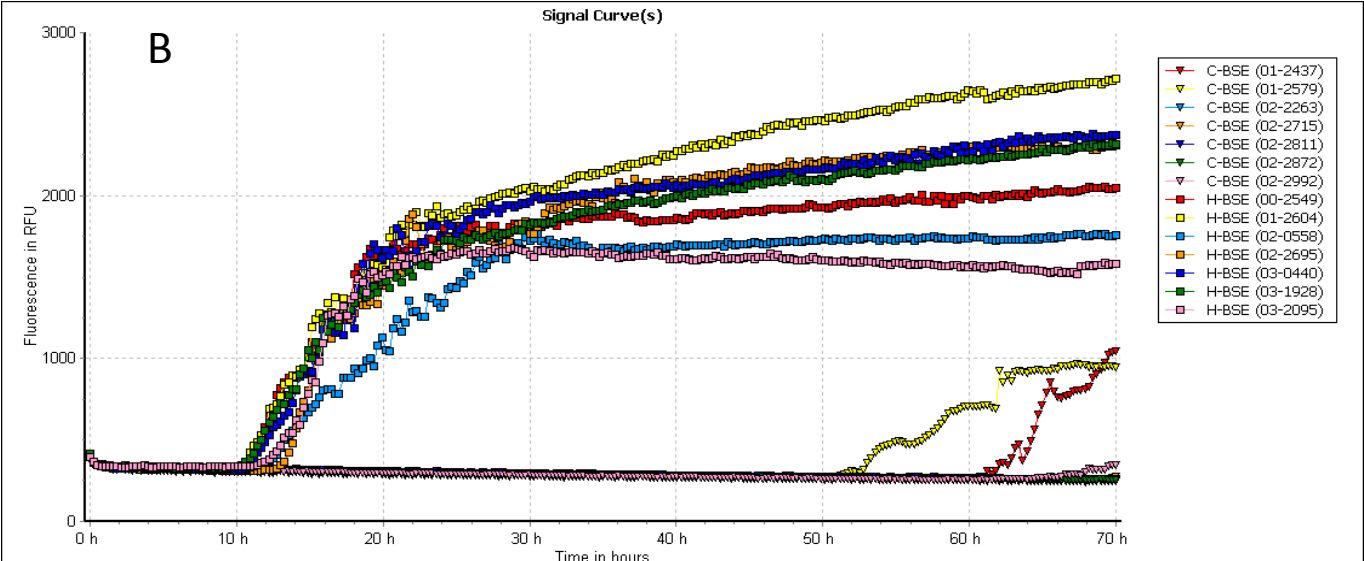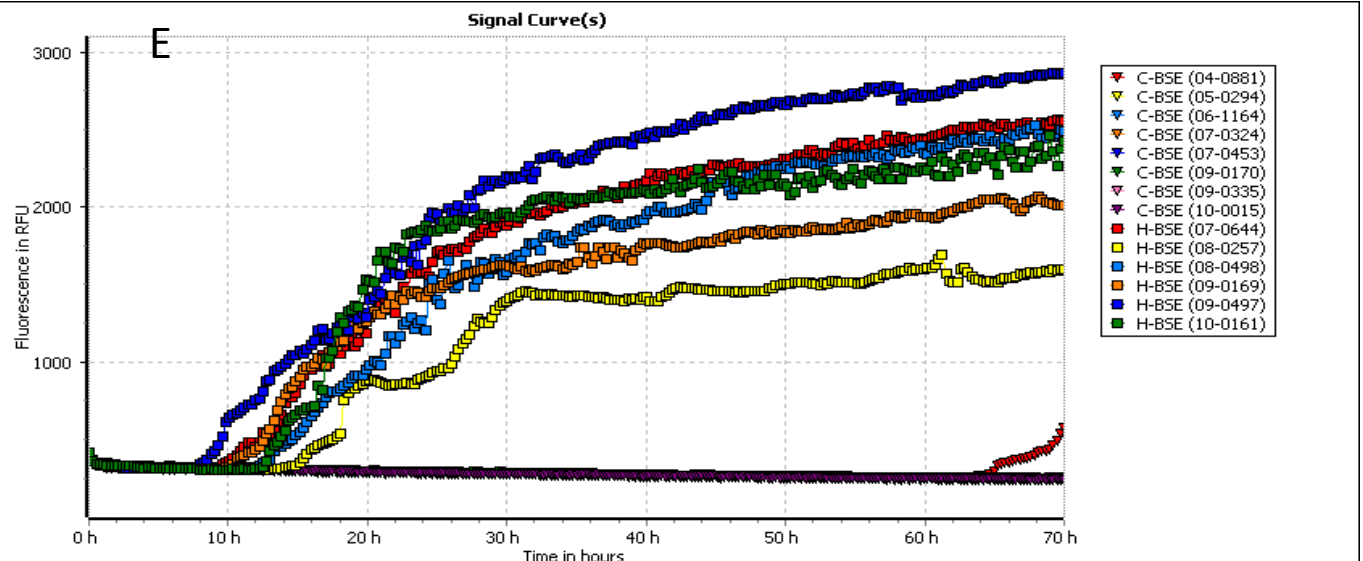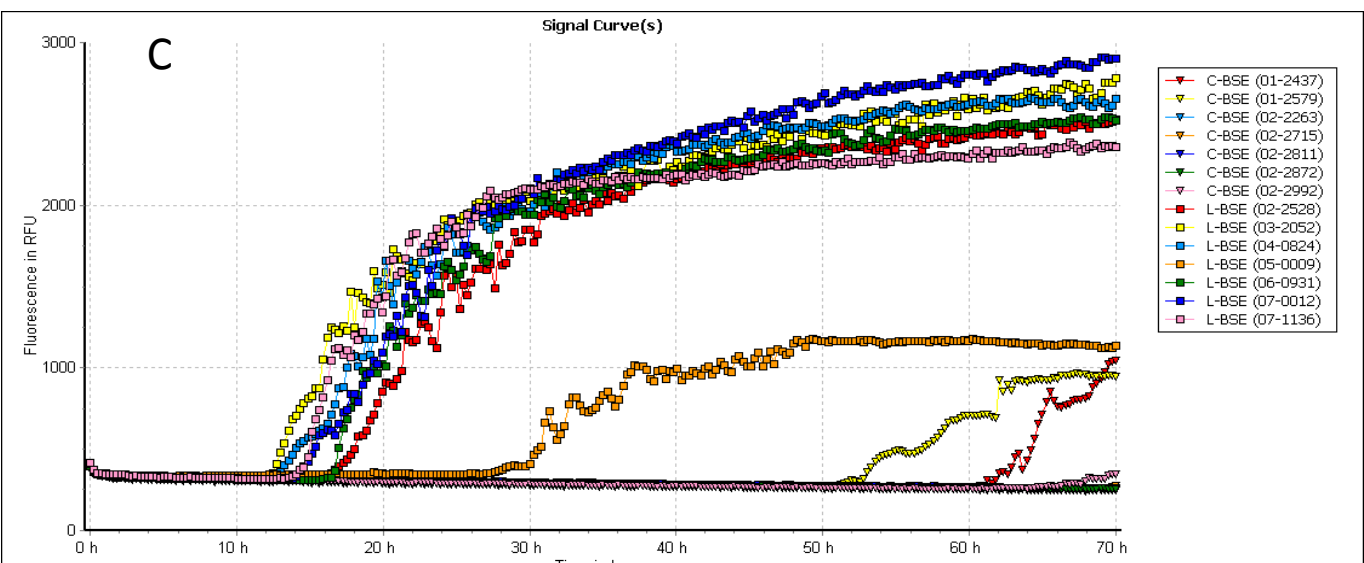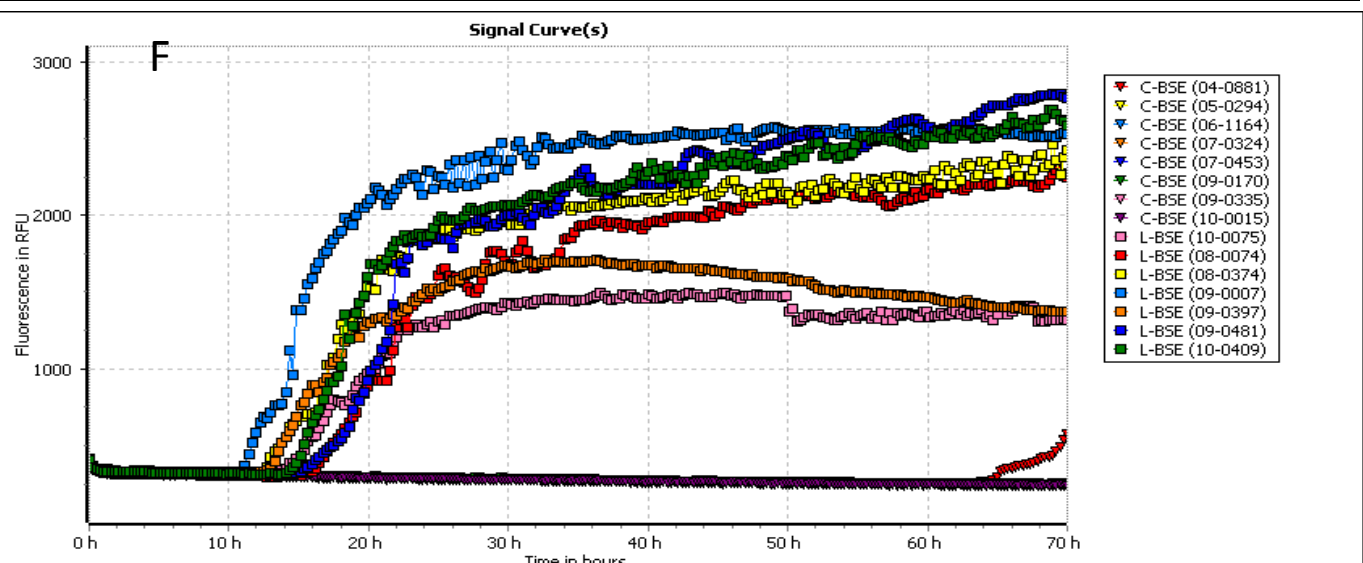

Supplement: S3 Fig — RT-QuIC reactions were seeded with 10−4 dilutions of bovine tissue (brainstem), using bovine recombinant protein. (A) and (D), classical BSE isolates and uninfected bovine samples. (B) and (E), classical BSE and atypical H-BSE isolates. (C) and (F), classical BSE and atypical L-BSE isolates. Each point represents the mean value of 3 replicate relative fluorescence unit readings. (PDF) [file pone.0172428.s003.pdf]

S4 Fig

A

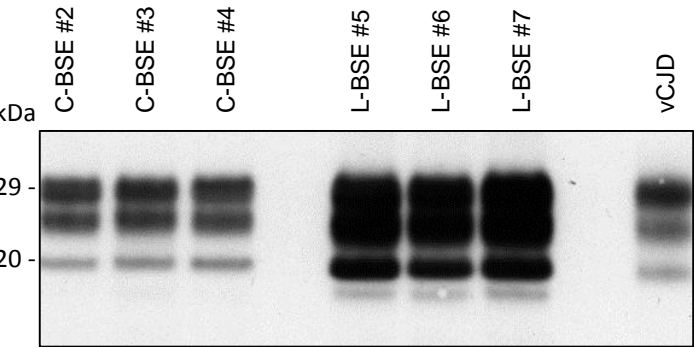

B

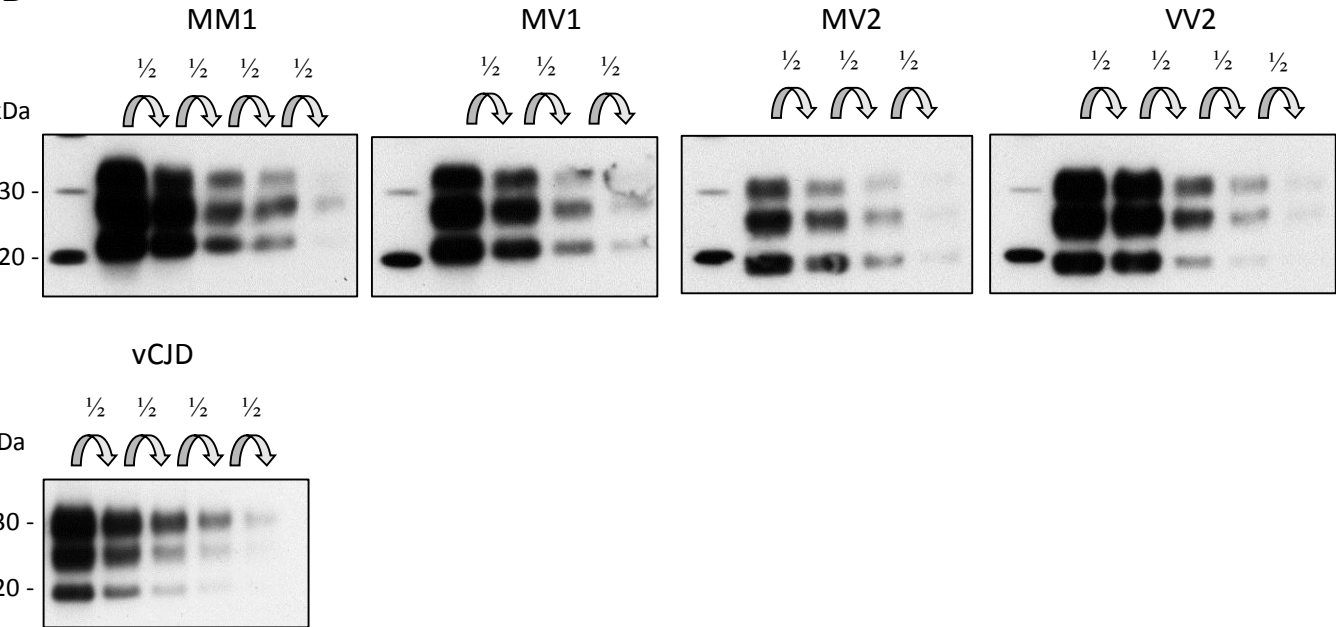

Supplement: S4 Fig — Homogenates were subjected to proteinase K digestion and serial dilutions were detected by immunoblotting using Sha31 (primates) or 3F4 (CJD patients) monoclonal antibodies. (PDF) [file pone.0172428.s004.pdf]
